# Tris(1,2-diamino­ethane)­nickel(II) hexa­fluoridosilicate

**DOI:** 10.1107/S1600536810041553

**Published:** 2010-10-23

**Authors:** Jaroslava Haníková, Juraj Kuchár, Juraj Černák, Giorgio Pelosi

**Affiliations:** a Department of Inorganic Chemistry, Institute of Chemistry, P. J. Šafárik University, Moyzesova 11, 041 54 Košice, Slovakia; b Dipartimento di Chimica Generale ed Inorganica, Universitá di Parma, Viale della Scienze, 43124 Parma, Italy

## Abstract

The ionic title complex, [Ni(C_2_H_8_N_2_)_3_](SiF_6_), is built up of [Ni(*en*)_3_]^2+^ complex cations (*en* = 1,2-diamino­ethane) and hexa­fluoridosilicate anions. Single crystals of the title complex were isolated from an aqueous–ethano­lic Ni^2+^–*en*–SiF_6_^2−^ system. The Ni(II) and Si atoms are each located on a special position with site symmetry 3.2. The Ni(II) atom coordination sphere is octa­hedrally deformed, being coordinated by three chelating diamine ligands with an Ni—N distance of 2.1233 (18) Å. The crystal packing of the respective ions corresponds to the structure type of the hexa­gonal form of BN. Beside ionic forces, the packing is governed by N—H⋯F hydrogen bonds, which lead to the formation of hydro­phobic channels running along the 6_3_ screw axis. The structure was refined as an inversion twin [0.49 (3): 0.51 (3)].

## Related literature

For the hexa­fluoridosilicate anion acting as simple counter-ion, see: Li *et al.* (2009 ▶). For two nickel(II) complexes containing the hexa­fluoridosilicate anion as counter-ion, see: Spek *et al.* (1988 ▶); Wu *et al.* (2008 ▶). For complexes containing the [Ni(*en*)_3_]^2+^ complex cation and hexa­fluorido-type anions, see: Pan *et al.* (2005 ▶); Ribas *et al.* (1998 ▶); James *et al.* (1998 ▶); Contakes *et al.* (2000 ▶). 
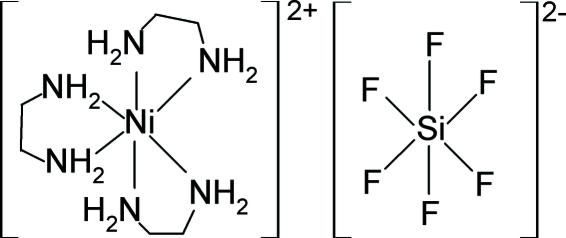


## Experimental

### 

#### Crystal data

[Ni(C_2_H_8_N_2_)_3_](SiF_6_)
*M* *_r_* = 381.11
Hexagonal, 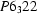

*a* = 9.1670 (9) Å
*c* = 9.763 (1) Å
*V* = 710.51 (12) Å^3^
*Z* = 2
Mo *K*α radiation
μ = 1.52 mm^−1^
*T* = 291 K
0.42 × 0.21 × 0.15 mm

#### Data collection

Oxford Diffraction Xcalibur diffractometer with Sapphire2 detector
Absorption correction: numerical [Clark & Reid (1995 ▶) in *CrysAlis PRO* (Oxford Diffraction, 2009 ▶)] *T*_min_ = 0.834, *T*_max_ = 0.859
8628 measured reflections
554 independent reflections
489 reflections with *I* > 2σ(*I*)
*R*_int_ = 0.050

#### Refinement

*R*[*F* ^2^ > 2σ(*F* ^2^)] = 0.027
*wR*(*F* ^2^) = 0.064
*S* = 1.07
554 reflections
33 parameters
H-atom parameters constrained
Δρ_max_ = 0.68 e Å^−3^
Δρ_min_ = −0.19 e Å^−3^
Absolute structure: Flack (1983 ▶), 89 Friedel pairs
Flack parameter: 0.49 (3)

### 

Data collection: *CrysAlis PRO* (Oxford Diffraction, 2009 ▶); cell refinement: *CrysAlis PRO*; data reduction: *CrysAlis PRO*; program(s) used to solve structure: *SHELXS97* (Sheldrick, 2008 ▶); program(s) used to refine structure: *SHELXL97* (Sheldrick, 2008 ▶); molecular graphics: *DIAMOND* (Crystal Impact, 2007 ▶); software used to prepare material for publication: *SHELXL97*.

## Supplementary Material

Crystal structure: contains datablocks I, Global. DOI: 10.1107/S1600536810041553/su2212sup1.cif

Structure factors: contains datablocks I. DOI: 10.1107/S1600536810041553/su2212Isup2.hkl

Additional supplementary materials:  crystallographic information; 3D view; checkCIF report

## Figures and Tables

**Table 1:** Hydrogen-bond geometry (Å, °)

*D*—H⋯*A*	*D*—H	H⋯*A*	*D*⋯*A*	*D*—H⋯*A*
N1—H1⋯F1^i^	0.90	2.30	3.137 (2)	154
N1—H1⋯F1^ii^	0.90	2.48	3.235 (2)	142
N1—H2⋯F1^iii^	0.90	2.25	3.137 (2)	167

Symmetry codes: (i) 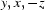; (ii) 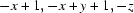; (iii) 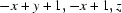.

**Fig. 1.**
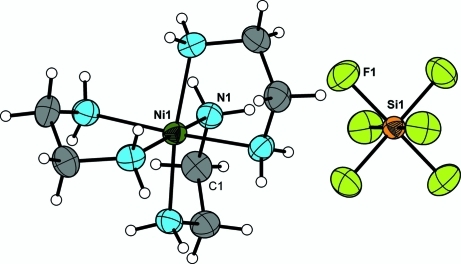
Molecular structure of the title ionic complex along with the atom numbering scheme. Displacement ellipsoids are drawn at the 50% probability level.

**Fig. 2.**
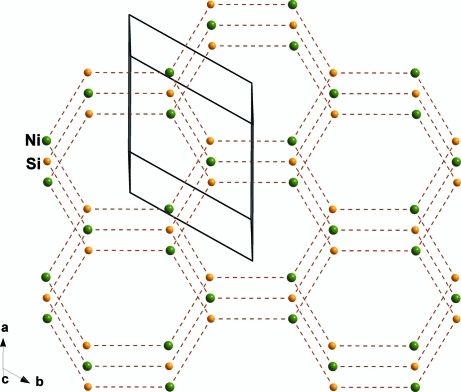
Packing of the ions in the title complex leading to the hexagonal BN structure type.

**Fig. 3.**
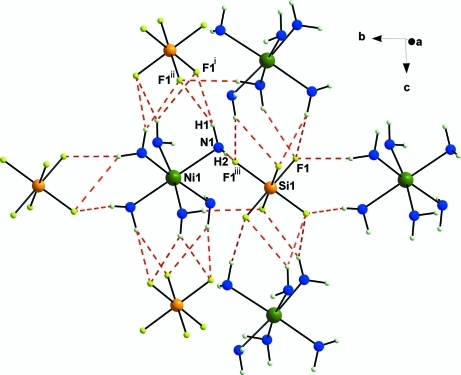
Detailed view of the hydrogen bonding scheme (dashed lines) in the title complex. For the sake of clarity only the NH2 groups of the en ligands are shown. Symmetry codes: (i) = y, x, -z; (ii) = 1 - x, 1 - x + y, z; (iii) = 1 - x + y, 1 - x, z.

**Fig. 4.**
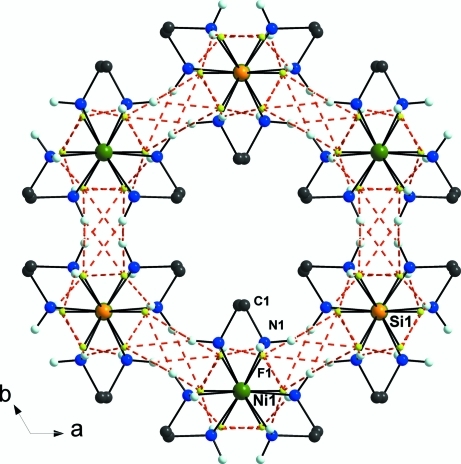
Formation of hydrophobic pseudo-channels inside the hydrogen bonded complex cations and anions running along the 63 screw axis: view of one channel. Hydrogen atoms bonded to the carbon atoms are omitted for the sake of clarity.

**Fig. 5.**
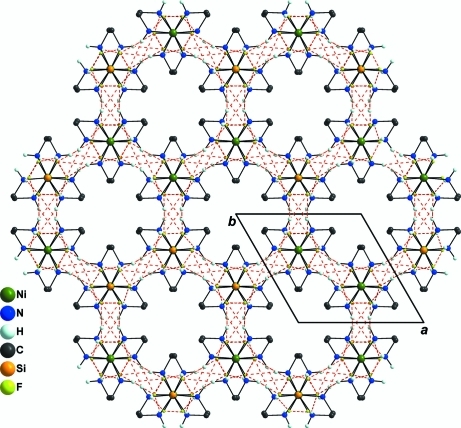
Formation of hydrophobic pseudo-channels inside the hydrogen bonded complex cations and anions running along the 63 screw axis: arrangement of the neighbouring channels. Hydrogen atoms bonded to the carbon atoms are omitted for the sake of clarity.

## supplementary crystallographic information

### Comment

The crystal structure of the title complex is ionic and is built up of [Ni(en)_3_]^2+^ complex cations and SiF_6_^2-^ anions, as shown in Fig. 1. The Ni^II^ atom (site symmetry 32) in the [Ni(en)_3_]^2+^ complex cation has a slightly deformed octahedral coordination sphere, being coordinated by six nitrogen atoms from three chelate bonded *en* ligands.

As the studied single-crystal was an inversion twin [ratio of the two domains was 0.49 (3):0.51 (3)] both Λδδδ and Δλλλ configurations were present in the crystal. In the isostructural [Zn(*en)*)_3_]SiF_6_ complex the cations exhibit Λδδδ absolute configuration (Li *et al.*, 2009). The Ni—N bond lengths of 2.1234 (18) Å (6 ×) corresponds well to the value of 2.1318 (2) Å found in the analogous hexafluoridogermanate complex [Ni(*en*)_3_]GeF_6_ (Pan *et al.*, 2005). The positive charge of the complex cation is compensated for by the non-coordinated SiF_6_^2-^ anion, that exhibits almost ideal octahedral symmetry. The Si atom is located on the 3-fold axis (site symmetry 32). The Si—F bond length of 1.681 (2) Å (6 ×) is in line with the value of 1.6942 (15) Å found in [Zn(*en*)_3_]SiF_6_ (Li *et al.*, 2009).

In the crystal the packing of the respective ions corresponds to the hexagonal structure of BN, with a Ni···Si distance of 5.2927 (4) Å within the hexagonal plane and a Ni···Si distance of 4.8815 (5) Å between the planes (Fig. 2). To the packing forces contribute also N—H···F type hydrogen bonds with N···F distances in the range 3.137 (2) - 3.235 (2) Å (Table 1, Fig. 3). Some of the hydrogen bonds are three-centered with two fluorido acceptors. The observed geometric parameters associated with the hydrogen bonds correspond to those in Zn(*en)*)_3_]SiF_6_ (Li *et al.*, 2009) where the N···F distances range from 3.113 (3) - 3.239 (3) Å. The hydrogen bonding leads to the formation of hydrophobic channels running along the 6_3_ screw axis (Fig. 4a and 4 b), as was already observed in the GeF_6_ analog (Pan *et al.*, 2005).

### Experimental

To a solution of 0.24 g of NiCl_2_.6H_2_O (1 mmol) in 10 cm^3^ of water:ethanol mixture (1:1 in vol) wre added successively 0.27 cm^3^ of 1,2-diaminoethane (en) (4 mmol) and 0.18 g of (NH_4_)SiF_6_ (1 mmol), dissolved in 10 cm^3^ of water:ethanol mixture (1:1 / v:v), under constant stirring. The dark pink solution that formed was filtered and left aside for crystallization at RT. Within a few days light-pink prisms were formed. They were collected by filtration and subsequently recrystallized from a water:ethanol mixture to give crystals suitable for X-ray diffraction analysis. Anal. [%], calculated for Ni_1_C_6_N_6_H_24_Si_1_F_6_: C, 18.92; H, 6.35; N, 22.05. Found: C, 18.97; H, 5.76; N, 14.55. IR (KBr pellets, FT—IR Avatar 330 (ThermoNicolet), cm^-1^): 3300*m*; 3170*m*; 2954*m*; 2925*m*; 2887*m*; 1598 s; 1456 s; 1385w; 1370w; 1125 s; 1064 s, 717 s; 500 s; 478*m*. Thermal Analysis (TA Instrument, air atmosphere): the complex was thermally stable up to 501 K and decomposed in one step in the temperature range 501 - 693 K.

### Refinement

The structure was refined as an inversion twin [0.49 (3): 0.51 (3)]. All the H atoms were included in calculated positions and treated as riding atoms: N—H = 0.90 Å, C—H = 0.97 Å, with *U*_iso_(H) = 1.2*U*_eq_(parent N– or C-atom).

### Crystal data

**Table:**

[Ni(C_2_H_8_N_2_)_3_](SiF_6_)	*D*_x_ = 1.781 Mg m^−^^3^
*M**_r_* = 381.11	Mo *K*α radiation, λ = 0.71069 Å
Hexagonal, *P*6_3_22	Cell parameters from 8628 reflections
Hall symbol: P 6c 2c	θ = 2.6–27.4°
*a* = 9.1670 (9) Å	µ = 1.52 mm^−^^1^
*c* = 9.763 (1) Å	*T* = 291 K
*V* = 710.51 (12) Å^3^	Prism, pink
*Z* = 2	0.42 × 0.21 × 0.15 mm
*F*(000) = 396	

### Data collection

**Table:**

Oxford Diffraction Xcalibur diffractometer with Sapphire2 detector	554 independent reflections
Radiation source: fine-focus sealed tube	489 reflections with *I* > 2σ(*I*)
graphite	*R*_int_ = 0.050
Detector resolution: 8.3438 pixels mm^-1^	θ_max_ = 27.4°, θ_min_ = 2.6°
ω scans	*h* = −11→11
Absorption correction: numerical [Clark & Reid (1995) in *CrysAlis PRO* (Oxford Diffraction, 2009)]	*k* = −11→11
*T*_min_ = 0.834, *T*_max_ = 0.859	*l* = −12→12
8628 measured reflections	

### Refinement

**Table:**

Refinement on *F*^2^	Secondary atom site location: difference Fourier map
Least-squares matrix: full	Hydrogen site location: inferred from neighbouring sites
*R*[*F*^2^ > 2σ(*F*^2^)] = 0.027	H-atom parameters constrained
*wR*(*F*^2^) = 0.064	*w* = 1/[σ^2^(*F*_o_^2^) + (0.0413*P*)^2^] where *P* = (*F*_o_^2^ + 2*F*_c_^2^)/3
*S* = 1.07	(Δ/σ)_max_ < 0.001
554 reflections	Δρ_max_ = 0.68 e Å^−^^3^
33 parameters	Δρ_min_ = −0.19 e Å^−^^3^
0 restraints	Absolute structure: Flack (1983), 89 Friedel pairs
Primary atom site location: structure-invariant direct methods	Flack parameter: 0.49 (3)

### Special details

**Table:**

Geometry. All e.s.d.'s (except the e.s.d. in the dihedral angle between two l.s. planes) are estimated using the full covariance matrix. The cell e.s.d.'s are taken into account individually in the estimation of e.s.d.'s in distances, angles and torsion angles; correlations between e.s.d.'s in cell parameters are only used when they are defined by crystal symmetry. An approximate (isotropic) treatment of cell e.s.d.'s is used for estimating e.s.d.'s involving l.s. planes.
Refinement. Refinement of *F*^2^ against ALL reflections. The weighted *R*-factor *wR* and goodness of fit *S* are based on *F*^2^, conventional *R*-factors *R* are based on *F*, with *F* set to zero for negative *F*^2^. The threshold expression of *F*^2^ > σ(*F*^2^) is used only for calculating *R*-factors(gt) *etc*. and is not relevant to the choice of reflections for refinement. *R*-factors based on *F*^2^ are statistically about twice as large as those based on *F*, and *R*- factors based on ALL data will be even larger.

### Fractional atomic coordinates and isotropic or equivalent isotropic displacement parameters (Å^2^)

**Table:**

	*x*	*y*	*z*	*U*_iso_*/*U*_eq_	
Ni1	0.3333	0.6667	0.2500	0.02783 (19)	
N1	0.3156 (2)	0.4642 (2)	0.13146 (17)	0.0368 (4)	
H1	0.3098	0.4839	0.0419	0.044*	
H2	0.4076	0.4547	0.1449	0.044*	
C1	0.1634 (3)	0.3071 (2)	0.1729 (2)	0.0432 (5)	
H5	0.1732	0.2107	0.1455	0.052*	
H6	0.0651	0.2992	0.1284	0.052*	
Si1	0.6667	0.3333	0.2500	0.0267 (3)	
F1	0.51790 (19)	0.18375 (19)	0.14983 (14)	0.0550 (4)	

### Atomic displacement parameters (Å^2^)

**Table:**

	*U*^11^	*U*^22^	*U*^33^	*U*^12^	*U*^13^	*U*^23^
Ni1	0.0286 (2)	0.0286 (2)	0.0262 (3)	0.01432 (11)	0.000	0.000
N1	0.0401 (10)	0.0429 (11)	0.0319 (9)	0.0241 (9)	0.0020 (8)	−0.0013 (8)
C1	0.0428 (16)	0.0339 (10)	0.0489 (12)	0.0162 (13)	−0.0016 (11)	−0.0079 (8)
Si1	0.0270 (3)	0.0270 (3)	0.0262 (5)	0.01349 (17)	0.000	0.000
F1	0.0521 (8)	0.0495 (8)	0.0489 (8)	0.0145 (6)	−0.0110 (7)	−0.0086 (7)

### Geometric parameters (Å, °)

**Table:**

Ni1—N1^i^	2.1233 (18)	C1—C1^iv^	1.515 (4)
Ni1—N1^ii^	2.1233 (18)	C1—H5	0.9700
Ni1—N1^iii^	2.1233 (18)	C1—H6	0.9700
Ni1—N1^iv^	2.1233 (18)	Si1—F1^vi^	1.6812 (14)
Ni1—N1	2.1233 (18)	Si1—F1^vii^	1.6812 (14)
Ni1—N1^v^	2.1233 (18)	Si1—F1	1.6812 (14)
N1—C1	1.475 (2)	Si1—F1^v^	1.6812 (14)
N1—H1	0.9000	Si1—F1^viii^	1.6812 (14)
N1—H2	0.9000	Si1—F1^ix^	1.6812 (14)
			
N1^i^—Ni1—N1^ii^	81.62 (9)	N1—C1—C1^iv^	109.08 (16)
N1^i^—Ni1—N1^iii^	93.12 (7)	N1—C1—H5	109.9
N1^ii^—Ni1—N1^iii^	92.62 (10)	C1^iv^—C1—H5	109.9
N1^i^—Ni1—N1^iv^	92.62 (10)	N1—C1—H6	109.9
N1^ii^—Ni1—N1^iv^	93.12 (7)	C1^iv^—C1—H6	109.9
N1^iii^—Ni1—N1^iv^	172.42 (9)	H5—C1—H6	108.3
N1^i^—Ni1—N1	93.12 (7)	F1^vi^—Si1—F1^vii^	90.75 (10)
N1^ii^—Ni1—N1	172.42 (9)	F1^vi^—Si1—F1	90.12 (10)
N1^iii^—Ni1—N1	93.12 (6)	F1^vii^—Si1—F1	89.57 (7)
N1^iv^—Ni1—N1	81.62 (9)	F1^vi^—Si1—F1^v^	89.57 (7)
N1^i^—Ni1—N1^v^	172.42 (9)	F1^vii^—Si1—F1^v^	90.12 (10)
N1^ii^—Ni1—N1^v^	93.12 (7)	F1—Si1—F1^v^	179.56 (10)
N1^iii^—Ni1—N1^v^	81.62 (9)	F1^vi^—Si1—F1^viii^	89.57 (7)
N1^iv^—Ni1—N1^v^	93.12 (6)	F1^vii^—Si1—F1^viii^	179.56 (10)
N1—Ni1—N1^v^	92.62 (10)	F1—Si1—F1^viii^	90.75 (10)
C1—N1—Ni1	108.97 (13)	F1^v^—Si1—F1^viii^	89.57 (7)
C1—N1—H1	109.9	F1^vi^—Si1—F1^ix^	179.56 (10)
Ni1—N1—H1	109.9	F1^vii^—Si1—F1^ix^	89.57 (7)
C1—N1—H2	109.9	F1—Si1—F1^ix^	89.57 (7)
Ni1—N1—H2	109.9	F1^v^—Si1—F1^ix^	90.75 (10)
H1—N1—H2	108.3	F1^viii^—Si1—F1^ix^	90.12 (10)
			
N1^i^—Ni1—N1—C1	−78.08 (18)	N1^v^—Ni1—N1—C1	106.88 (16)
N1^iii^—Ni1—N1—C1	−171.38 (15)	Ni1—N1—C1—C1^iv^	−39.4 (3)
N1^iv^—Ni1—N1—C1	14.11 (12)		

Symmetry codes: (i) −*x*+*y*, −*x*+1, *z*; (ii) *x*, *x*−*y*+1, −*z*+1/2; (iii) −*y*+1, *x*−*y*+1, *z*; (iv) −*x*+*y*, *y*, −*z*+1/2; (v) −*y*+1, −*x*+1, −*z*+1/2; (vi) −*x*+*y*+1, *y*, −*z*+1/2; (vii) −*y*+1, *x*−*y*, *z*; (viii) *x*, *x*−*y*, −*z*+1/2; (ix) −*x*+*y*+1, −*x*+1, *z*.

### Hydrogen-bond geometry (Å, °)

**Table:**

*D*—H···*A*	*D*—H	H···*A*	*D*···*A*	*D*—H···*A*
N1—H1···F1^x^	0.90	2.30	3.137 (2)	154
N1—H1···F1^xi^	0.90	2.48	3.235 (2)	142
N1—H2···F1^ix^	0.90	2.25	3.137 (2)	167

Symmetry codes: (x) *y*, *x*, −*z*; (xi) −*x*+1, −*x*+*y*+1, −*z*; (ix) −*x*+*y*+1, −*x*+1, *z*.

## References

[bb1] Clark, R. C. & Reid, J. S. (1995). *Acta Cryst.* A**51**, 887–897.

[bb2] Contakes, S. M., Klausmeyer, K. K. & Rauchfuss, T. B. (2000). *Inorg. Chem.* **39**, 2069–2075. 10.1021/ic991037r 12526514

[bb3] Crystal Impact (2007). *DIAMOND* Crystal Impact, Bonn, Germany.

[bb4] Flack, H. D. (1983). *Acta Cryst.* A**39**, 876–881.

[bb5] James, M., Kawaguchi, H. & Tatsumi, K. (1998). *Polyhedron*, **17**, 1571–1577.

[bb6] Li, Y., Shi, Q., Slawin, A. M. Z., Woollins, J. D. & Dong, J. (2009). *Acta Cryst.* E**65**, m1522. 10.1107/S1600536809045693 PMC2972015 21578568

[bb7] Oxford Diffraction (2009). *CrysAlis PRO* Oxford Diffraction Ltd, Abingdon, England.

[bb8] Pan, Q.-H., Li, J.-Y., Yu, J.-H., Wang, Y., Fang, Q.-R. & Xu, R.-R. (2005). *Gaodeng Xuexiao Huaxue Xuebao*, **26**, 2199–2200.

[bb9] Ribas, J., Monfort, M., Resino, I., Ghosh, B. K., Solans, X. & Font-Bardia, M. (1998). *Polyhedron*, **17**, 1735–1739.

[bb10] Sheldrick, G. M. (2008). *Acta Cryst.* A**64**, 112–122. 10.1107/S0108767307043930 18156677

[bb11] Spek, A. L., Duisenberg, A. J. M., Bouwman, E., Driessen, W. L. & Reedijk, J. (1988). *Acta Cryst.* C**44**, 1569–1572.

[bb12] Wu, L.-P., Zhao, S.-M., Zhang, G.-F. & Ng, S. W. (2008). *Acta Cryst.* E**64**, m802. 10.1107/S1600536808013329 PMC2961398 21202489

